# Domains of Housing Instability and Intimate Partner Violence Risk Among U.S. Tenants

**DOI:** 10.3390/ijerph22081212

**Published:** 2025-07-31

**Authors:** Anairany Zapata, Leila G. Wood, Annalynn M. Galvin, Wenyaw Chan, Timothy A. Thomas, Jack Tsai, Heather K. Way, Elizabeth J. Mueller, Daphne C. Hernandez

**Affiliations:** 1 Cizik School of Nursing, University of Texas Health Science Center at Houston, Houston, TX 77030, USA; anairany.zapata@uth.tmc.edu (A.Z.); annalynn.m.galvin@uth.tmc.edu (A.M.G.); 2 McGovern Medical School, University of Texas Health Science Center at Houston, Houston, TX 77030, USA; leila.g.wood@uth.tmc.edu; 3 School of Public Health, University of Texas Health Science Center at Houston, Houston, TX 77030, USA; wenyaw.chan@uth.tmc.edu (W.C.); jack.tsai@uth.tmc.edu (J.T.); 4 Institute of Governmental Studies, University of California, Berkeley, CA 94720, USA; timthomas@berkeley.edu; 5 School of Law, The University of Texas at Austin, Austin, TX 78705, USA; hway@law.utexas.edu; 6 School of Architecture, The University of Texas at Austin, Austin, TX 78712, USA; ejmueller@austin.utexas.edu

**Keywords:** domestic violence, homelessness, housing insecurity, housing instability, IPV, lease violations, unsafe housing environment, utility hardship

## Abstract

While IPV is often studied as a predictor of housing insecurity, few U.S. studies explore how different forms of housing instability may contribute to intimate partner violence (IPV) risk. Using a mixed-methods approach and a cross-sectional design, this study examined the association between four housing instability domains and IPV among a sample of tenants that had either experienced eviction or were at high risk for eviction. Tenants in Harris and Travis counties (Texas, USA) completed an online survey (*n* = 1085; March–July 2024). Housing instability was assessed across four domains: homelessness, lease violations, utility hardship, and poor housing quality. IPV was measured using the Hurt, Insult, Threaten, Scream Screener. Covariate-adjusted logistic regression models suggest indicators within the four housing instability domains were associated with IPV risk. Within the homelessness domain, experiences with lifetime homelessness (AOR = 1.92, 95%CI 1.61–2.28), in the past 12 months living in unconventional spaces (AOR = 2.10, 95%CI 1.92–2.29), and moving in with others (AOR = 1.20, 95%CI 1.06–1.36) were associated with IPV. Within the lease violations domain, missed rent payments (AOR = 1.69, 95%CI 1.68–1.71) and non-payment lease violations (AOR = 2.50, 95%CI 2.29–2.73) in the past 12 months were associated with IPV. Utility shutoffs (AOR = 1.62, 95%CI 1.37–1.91) and unsafe housing (AOR = 1.65, 95%CI 1.31–2.09) in the past 12 months were associated with IPV. Homelessness, housing-related economic hardships and substandard living conditions predict an elevated risk of IPV.

## 1. Introduction

Intimate partner violence (IPV), or behavior with a spouse or romantic partner that causes physical, sexual, or psychological harm [1], is experienced by over 40% of adults in the U.S. [2] and increased by 8% during the stay-at-home period of the 2020 COVID-19 pandemic [3]. Globally, almost one-third of women aged 15–49 have experienced intimate partner physical and/or sexual violence [4]. U.S. women who experienced IPV in the past year were four times more likely to experience housing instability compared to women who did not experience IPV [5]. Housing instability is generally defined as the state of living in housing but currently being at-risk of losing that housing [6]. In other words, it is the extent to which quality housing is insecure [7]. Housing instability is commonly measured as experiencing an eviction or homelessness. Among U.S. and Canadian women, past literature establishes the interconnectedness of IPV and eviction and homelessness [8,9,10,11,12,13,14]. For example, 20 to 38% of women who have experienced homelessness have also experienced IPV [8,13]. Similarly, one study reported 20% of women who were evicted experienced IPV, with the IPV frequently occurring before they were first evicted [9].

Housing instability can also include more finite measures of homelessness, such as housing mobility (i.e., frequent housing transitions), living in emergency shelters or transitional housing, living in places not meant for residence (e.g., in cars, outdoors), and doubling up (i.e., living with others due to financial strain). For example, adults who experience IPV may experience frequent moves within short amounts of time to escape their abuse [15]. When IPV occurs, the most common community-based housing accommodation in the U.S. is a shelter [16]. Shelters are temporary housing facilities for individuals experiencing homelessness and are often at capacity and under-resourced, making it difficult for all individuals in need of accommodation [17]. Those who do not reside in shelters may live in other unconventional spaces, such as an abandoned building or a car, or stay in unsafe housing with a violent partner.

Adults who experience housing instability may have difficulty maintaining a residential dwelling as a consequence of experiencing housing-related economic hardships. For instance, U.S. adults who experience IPV are also more likely to experience lease and utility hardships, such as difficulties paying for rent and utilities and disconnected phone service [10,18]. Within lease violations, research has mainly focused on non-payment of rent and not made the distinction between paying a partial amount of rent and missing the entire rent payment with the risk for IPV experiences [10,18]. Further, in the U.S. violations of lease contracts can also be related to other behaviors unrelated to payment, such as breaking a lease agreement [e.g., owning an unauthorized pet, subletting the property without permission, property damage, illegal activity (e.g., substance abuse)] or a nuisance violation, which involves disorderly conduct interfering with other tenants enjoying their homes (e.g., noise complaints from music, parties, barking dogs, shouting) [19]; yet non-payment related violations have not been examined in relation to IPV experiences.

Maintaining a residential dwelling may also be difficult due to environmental factors. The quality of rental dwellings is measured through indicators assessing various housing conditions, including exposure to pests (e.g., cockroaches and rats) and mold. Indicators of poor housing conditions—such as living in housing with pests and mold—has not been previously examined in association with IPV among U.S. samples. An additional indicator of housing quality is neighborhood safety [10]. U.S. adults who experience IPV, in addition to experiencing violence within the household, also have a greater likelihood of living in neighborhoods with higher levels of social disorder, disadvantage, and community violence [20,21], which may relate to IPV victimization among women [21].

Research that has primarily occurred in the U.S. has focused on the relationship between IPV and housing insecurity—including eviction and homelessness [8,9,10,11,12,13,14] and housing mobility [15]. While some research has been conducted on nonpayment of rent and utilities [10,18] and unsafe housing conditions [10,20,21], research is lacking on measures of lease violations and poor living conditions. The current U.S.-based study collected an abundance of measures of housing instability providing the opportunity to create four domains of housing instability. By focusing on four domains of housing instability—homelessness, lease violations, utility hardship, and poor housing quality—this article aims to identify tenants at risk for IPV victimization according to the domains. The Family Stress Model links economic stress within the family unit to negative behavioral outcomes [22]. Resource Theory also takes a resource deficit approach and emphasizes how the imbalance of resources among couples could lead to violence [23]. Both the Family Stress Model and Resource Theory [22,23] support this article’s aim based on the premise that economic adversity enhances violence. Within romantic relationships, economic adversity increases psychological distress and conflict. The increase in distress and conflict places adults at risk for experiencing IPV [22,23], especially when coercive control strategies are already present. Coercive control theory [24] suggests that strategies, including monitoring and surveillance, are used to establish power and control [25], especially among the economically vulnerable [26]. This study contributes to the literature by including various measures of housing instability, including measures that have been traditionally excluded in identifying adults at risk for IPV. Most IPV studies that have focused on housing instability/homelessness have selected their sample from U.S. shelters and services dedicated to IPV [8,10,14,16]. This study is innovative in that sampling is derived from two sources of data: (1) court records that identify U.S. tenants who have experienced an eviction and (2) spatial machine learning models that identify U.S. adults who live in neighborhoods that are at highest risk for eviction.

## 2. Materials and Methods

### 2.1. Data and Sample

The sample was derived from the Health Outcomes Post-Eviction-Moratoria (HOPE-M) study, which is a mixed-methods study designed to assess the impact of eviction on mental health among tenants residing in Harris and Travis Counties (Texas, USA) after the COVID-19 eviction moratoria ended. The HOPE-M study comprises a sample of renters who have experienced a formal eviction (“the evicted sample”) and a sample of renters who reside in a neighborhood at risk for eviction (“the non-evicted sample”). The evicted sample is derived from eviction records collected for analysis by a data science consulting firm, January Advisors, for the period between when COVID-19 state and local eviction moratoria ended through December 2023 (Harris: June 2020–December 2023; Travis: January 2022–December 2023).

The non-evicted sample for HOPE-M study was derived using the Housing Precarity Risk Model (HPRM) [27]. HPRM uses spatial machine learning techniques to facilitate identification of tenants living in neighborhoods with high eviction risk after the state and local eviction moratoria ended. Tenants were selected using the same timeframe as the evicted sample but had not experienced an eviction during this period. In addition, tenants were selected using criteria based on respondent household characteristics that align with the top predictive variables in the HPRM (e.g., race) as well as dominate characteristics found in prior eviction research (e.g., sex) [27,28].

**Figure 1:**
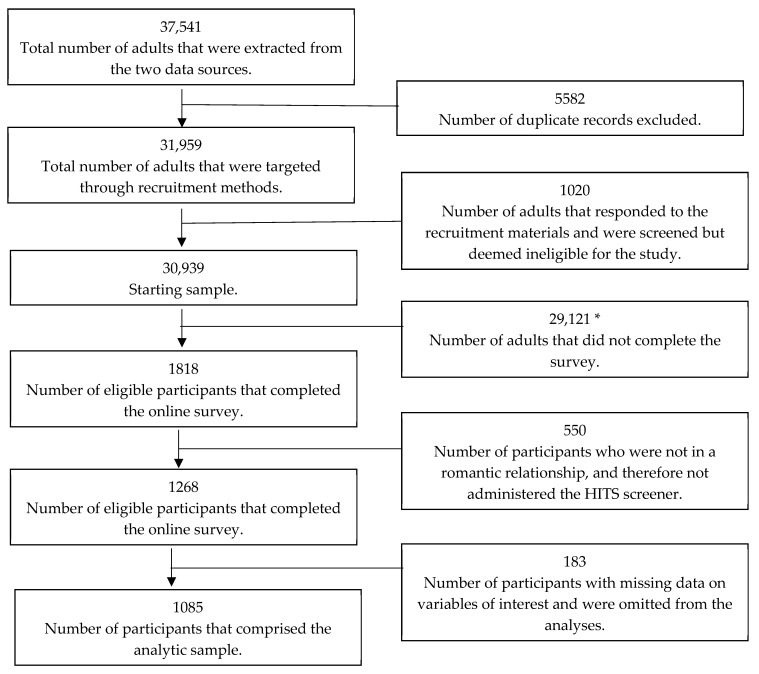
Sample selection process for the HOPE-M study and for the analytic sample. NOTE. Sample is derived from two data sources: court-ordered eviction records and the Housing Precarity Risk Model [26]. HPRM identifies individuals living in neighborhoods with high eviction risk using spatial machine learning techniques. The sample comprises tenants residing in Harris and Travis Counties (Texas). * This number may include individuals who were ineligible for the survey but did not respond to the recruitment screener, making their eligibility status unknown.

For the HOPE-M study, a total of 37,541 potential participants were extracted from the data sources (Figure 1). After removing duplicates, the recruitment sample began with 31,959 potential participants. Trestle software (https://trestleiq.com/) was then used to generate names, addresses, phone numbers, and emails [29]. Initial recruitment was conducted through a mailer, followed by email and text messages (when information was available). A contractor, M. Davis and Company, mailed a letter describing the purpose of the study, a list of frequently ask questions and responses in English and Spanish about the study and the survey, and a personalized link to the online survey. Recruitment mailers also included $1 non-contingent incentive. Non-contingent incentives have been shown to increase participation among non-compliers and consequently decrease non-response bias and increase response rates compared to not having an upfront unconditional payment [30,31]. In other words, non-contingent incentives increase the legitimacy of surveys among populations where fear or trust may be lacking. Prior to engaging in the online survey, all potential participants responded to a two-item economic hardship and two-item housing online screener. Affirmative responses to the screener questions were a requirement for survey eligibility (n = 1020 ineligible). Eligible participants then provided informed consent. Follow-up emails, text messages, and phone calls were sent to potential participants who had not completed the survey. A total of 1818 eligible participants completed the online survey from March–July 2024 (5.8% response rate). The response rate is in line with individual-level microdata from the Household Pulse Survey [32]. Eligible participants that completed the survey were compensated with a $35 electronic gift card. The study was approved by the Institutional Review Board of the University of Texas Health Science Center–Houston.

### 2.2. Measures

*Housing Instability*. Housing instability was assessed via four domains: homelessness, lease violations, utility hardship, and housing quality. The *homelessness* domain measured participant’s lifetime experiences with homelessness, evictions post-COVID-19 moratoria, as well as recent experiences (past 12 months) with various types of homelessness that included mobility, staying in a place not meant for regular housing (i.e., unconventional space), and moving in with others. Participants affirmatively responded if they had ever experienced homelessness as a child or adult, defined as doubling up with friends or family, staying in a shelter, in a car/RV, or outdoors because they lost their housing as an adult or child. Experiencing a formal eviction was based on court evictions records during the time period described in the sample selection above. Participants also provided information about various types of homelessness experiences in the past 12 months. Specifically, participants stated the number of times they moved from one house (or apartment) to another, and indicated whether they had stayed in a shelter, abandoned building, automobile, or a place not meant for regular housing (i.e., unconventional space), and whether they had moved in with others because of financial problems in the past 12 months.

The *lease violations* domain was assessed by an affirmative response to three mutually exclusive items: not paying the full amount of rent payments, missing any rent payments, or violating any part of lease that is not related to payment in the past 12 months.

*Utility hardship* was measured by affirmative response in the past 12 months to having any of following utilities turned off: gas, electricity, or water.

The *poor housing quality* domain was assessed by affirmative responses in the past 12 months to two mutually exclusive items: participants’ experiences with poor living conditions and the safety of their housing environment. Participants were asked if they had problems with eight mutually exclusive categories: pests, mold, lead paint/pipes, lack of air conditioning, lack of heat, malfunctioning oven or stove, missing/malfunctioning smoke detectors, or water leaks. The total number of responses were summed, with higher scores suggesting worse living conditions. Participants affirmatively responded if they felt unsafe where they currently live.

*Intimate Partner Violence.* IPV was measured using the four-item Hurt, Insult, Threaten, Scream (HITS) Screener [33]. Participants affirmatively reported on whether they had experienced being: (1) physically hurt, (2) insulted or talked down to, (3) threatened with harm, or (4) screamed or cursed at by their current spouse/partner/significant other. An affirmative response to experiencing any of the four items was considered an experience with IPV.

*Covariates*. Socio-demographic characteristics were included to control for factors that may be associated with housing hardship and IPV. Variables included age (years), sex (1 = male; 0 = female), race [White, Black (reference), Hispanic], education [less than high school graduate, some college or technical school (reference), college graduate], employment (1 = unemployed; 0 = employed), annual household income [$0–≤ $14,999, $15,000–$34,999, $35,000–$49,999, $50,000 or higher (reference)], utilization of a rental assistance program or voucher to help pay rent (1 = yes; 0 = no), marital status (1 = married; 0 = single), and household composition (number of children and number of adults).

### 2.3. Analytic Plan

Descriptive analyses were conducted over the full analytic sample and by IPV status. Bivariate analyses were performed to compare housing hardship and socio-demographic characteristics by IPV sub-samples using chi-squared tests for categorical variables and *t*-tests for continuous variables. A covariate-adjusted logistic regression was conducted on the full analytic sample to examine associations between housing hardship measures and IPV. Standard errors in the regression model were corrected by clustering on the county indicator variable to account for multiple observations within a county. All analyses were conducted using Stata software, version 16 [34].

## 3. Results

### 3.1. Sample Characteristics

Descriptive characteristics for the full analytic sample (n = 1085) and by IPV status are described in Table 1. Eleven percent (n = 117) of the participants reported experiencing IPV. The most frequent housing hardships experienced by participants included having not paid their full amount of rent (61%), having lived in an unsafe housing environment (59%) in the last 12 months, or having ever experienced homelessness (62%). Participants were 39 years of age on average (SD = 11.86), 76% were female, 55% were Black (54.65%), while 25% were Hispanic. In addition, 41% had some college education, 67% were employed, and 74% reported an average household income of less than $50,000. In general, individuals who experienced IPV were more likely to also experience various forms of homelessness, lease violations, utility hardship, and poor housing quality compared to individuals who did not experience IPV.

### 3.2. Associations Between Housing Instability Domains and IPV

Various measures of homelessness, lease violations, utility hardships, and poor housing quality were all significantly associated with IPV experiences. Under the domain of homelessness, having ever experienced homelessness in their lifetime (Adjusted Odds Ratio (AOR) = 1.92, 95% CI 1.61–2.28, *p* < 0.001), having lived in a place not meant for housing in the past 12 months (AOR = 2.10, 95% CI 1.92–2.29, *p* < 0.001), and having moved in with other people in the past 12 months (AOR = 1.20, 95%CI 1.06–1.36, *p* < 0.01) were all significantly associated with having experienced IPV (Table 2). Under lease violations, having missed rent payments in the past 12 months (AOR = 1.69, 95% CI 1.68–1.71, *p* < 0.001) and having violated any part of a lease (not related to payment) in the past 12 months (AOR = 2.50, 95% CI 2.29–2.73, *p* < 0.001) were significantly associated with IPV experience (Table 2). Having had utilities turned off in the past 12 months (AOR = 1.62, 95% CI: 1.37–1.91, *p* < 0.001) and having lived in an unsafe housing environment in the past 12 months (AOR = 1.65, 95% CI: 1.31–2.09, *p* < 0.001) were significantly associated with IPV experiences.

## 4. Discussion

This study examined the association between four housing instability domains and IPV among a sample of U.S. tenants that had either experienced eviction or were at high risk for eviction. Similarly to previous literature, various measures of homelessness, including lifetime experience with homelessness, and more recent experiences (i.e., in the last 12 months) with homelessness, such as living in a place not meant for regular housing (e.g., shelter, car/RV) and moving in with other people because of financial problems—were related to IPV experiences [8,10,12,13,14,16]. In other words, experiencing homelessness at any point in time (lifetime or in the last 12 months) places adults at risk for also experiencing IPV. Previous U.S.-based research has suggested that IPV victimization makes adults more vulnerable to experiencing housing instability [5,9,12]. It is recommended that future research investigate the mechanisms related to the association between lifetime experiences of homelessness and IPV. There could be adverse experiences, such as early and/or cumulative exposure to poverty, neglect, family or community violence, that may serve as the underlying mechanisms between lifetime experiences of homelessness and IPV [35]. In addition, it is recommended that future research examine the potential bidirectional relationship between various measures of homelessness and IPV victimization, as the relationship may be reciprocal.

In addition, several recent housing instability experiences within the lease violations and utility hardship domains were associated with reported IPV victimization experiences. Like prior U.S.- and Canadian-based research, missing rent payments and utilities being turned off were positively associated with experiencing IPV [10,18]. Also, the current study explored an often-overlooked housing hardship indicator and its relation to IPV: lease violations not related to payment. Lease violations unrelated to payment could be related to breaking a lease agreement or a nuisance violation [19]. The findings-missing rent payments, violating a lease unrelated to payment, and utilities turned off as predictors of IPV-are in line with the Family Stress Model and Resource Theory [22,23]. The stress associated with lease violations and utilities being turned off places couples in a resource deficit and/or potentially couples experience an imbalance of resources. The lack of resources or the imbalance of resources within the home environment may be underlying factors associated with IPV experiences.

Additionally, the findings suggest that there may be factors external to the couple’s control that perpetuate IPV experiences. This study found that an unsafe housing environment predicted IPV experiences, similar to previous studies [10,20,21]. While the substandard living conditions could reflect the landlord’s negligence and be out of the tenant’s control, the findings emphasize that the complex tension associated with economic adversity within and outside the home environment contributes to power and control and may influence IPV victimization risks. These findings also support coercive control theory, where financial abuse tactics, such as exploitation and monitoring, create economic insecurity and risks for unsafe and unstable housing [36].

### Strengths and Limitations

A strength of this study is its demographically diverse sample. Because the sample was derived using linked formal eviction records and spatial machine learning techniques during a period of economic adversity and housing precarity after the sunsetting of COVID-19 eviction moratoria, a variety of tenant experiences that are not regularly investigated in IPV research were captured. Additionally, the multiple and complex housing instability domains provide a better comprehensive understanding of how various measures of homelessness, housing-related economic hardships and substandard living conditions can be related to IPV risk. However, this study also had some limitations. First, because the data was collected at just one point in time, it is not possible to determine the causality between housing hardship and IPV experiences. Second, there is some ambiguity in a few of the measures. The safety measure did not explicitly state that safety concerns were about the neighborhood environment or the environment external to the tenant’s home. Based on qualitative data associated with this project, it appears that safety concerns may be related to the neighborhood, but it cannot be stated with certainty. While prior research suggests that a third of “nuisance” citations are related to IPV and that a greater proportion of women are affected by these citations [19], the “lease violation unrelated to payment” measure in the study does not indicate the type of lease violation. Further, the lease violation data is based on perception and not on administrative records. Thus, a warning might be mistakenly perceived as a formal violation, even though it was only intended as a caution. The IPV items were in relation to the current spouse/partner; yet the measures lacked specificity in pinpointing the specific timeframe (e.g., past 12 months). Thus, participants could be using different recall periods, introducing data inaccuracies. Because the focus of this study is on establishing a relationship and not necessarily establishing patterns or trends over time, this is less of a concern. IPV was also measured using the HITS screener [33]. HITS recognizes recent verbal and physical abuse but can possibly underrecognize other forms of IPV, including psychological, sexual or financial abuse. Lastly, while the sample was diverse, the sample was derived from only two urban counties in Texas. Thus, our findings may not be generalized to adults located in rural areas or areas with different housing markets or legal frameworks.

## 5. Conclusions

In sum, this study showed that a wide range of measures–experiences with lifetime and current homelessness, housing-related economic hardships, and substandard living conditions are linked to increased risk of IPV among tenants. The findings suggest that U.S. policies that address multiple forms of housing instability (e.g., homelessness, housing-related economic hardship) are needed to reduce and prevent IPV. To do this, a multi-faceted approach is needed. First, better identification of who may be at risk for either housing instability or IPV is needed. Integrating housing instability and IPV screeners that measure a diverse set of indicators as a part of annual physical exams (i.e., annual checkup or wellness visit) may help to pinpoint a greater number of U.S. adults at-risk either for housing instability, IPV, or both. The screeners could be completed as a part of pre-appointment paperwork, either at home or while sitting in the waiting room. This is similar to conducting a food insecurity screener during a child’s wellness visit, which is recommended by the American Academy of Pediatrics [37].

Second, programs designed to reduce economic hardship must work together. Greater coordinated effort between U.S.-based programs that provide financial assistance with basic needs (e.g., subsidized housing, Supplemental Nutrition Assistant Program, Low Income Home Energy Assistance Program) is needed. To assist with the coordination of programs, policies could be revised to consider automatic eligibility. This is where a household that is eligible for one program is automatically eligible for other programs that assist with economic hardship. Ideally, households that enroll in multiple programs will receive greater financial assistance. Policies could also consider linking required paperwork between programs. This will cut down on paperwork and may reduce the discrimination and shame that applicants experience when applying and participating in financial assistance programs [38,39]. Greater efforts placed on identifying adults at risk and coordination of anti-poverty programs may help to reduce housing instability and experience with IPV.

## Figures and Tables

**Table 1:** Descriptive statistics for the full analytic sample and by intimate partner violence (IPV) victimization experience.

	Full Sample (n = 1085)				Intimate Partner Violence Victimization (n = 117)				No Intimate Partner Violence Victimization (n = 968)				*p*-Value ^a^
	#	% or Mean	SD	Range	#	% or Mean	SD	Range	#	% or Mean	SD	Range	
*Intimate Partner Violence Victimization*													
IPV	117	10.78%			117	100.00%			-	-			-
No IPV	968	89.22%			-	-			968	100.00%			-
*Housing Instability*													
*Homelessness*													
Lifetime experience with homelessness	672	61.94%			96	82.05%			576	59.50%			*p* < 0.001
Experienced eviction post-COVID-19 moratoria ^b^	507	46.73%			62	52.99%			445	45.97%			*p* = 0.151
Housing mobility (i.e., # of moves) ^c^		0.84	1.20	0–12		1.04	1.17	0–5		0.81	1.20	0–12	*p* = 0.046
Shelter, abandoned building, car (i.e., unconventional space) ^c^	198	18.25%			50	42.74%			148	15.29%			*p* < 0.001
Moved in with other people ^c^	373	34.38%			62	52.99%			311	32.13%			*p* < 0.001
*Lease Violations* ^c^													
Not pay full amount of rent	667	61.47%			84	71.79%			583	60.23%			*p* = 0.015
Missed rent payments	517	47.65%			80	68.38%			437	45.14%			*p* < 0.001
Violated any part of lease (not related to payment)	107	9.86%			30	25.64%			77	7.95%			*p* < 0.001
*Utility Hardship* ^c^													
Utilities turned off	467	43.04%			73	62.39%			394	40.70%			*p* < 0.001
*Poor Housing Quality* ^c^													
Poor living conditions		1.30	1.30	0–4		1.77	1.33	0–4		1.25	1.29	0–4	*p* < 0.001
Unsafe housing environment	645	59.45%			91	77.78%			554	57.23%			*p* < 0.001
*Sociodemographic Controls*													
Age		39.24	11.86	20–82		37.49	10.57	20–67		39.45	11.99	20–82	*p* = 0.170
Sex													
Male	265	24.42%			39	33.33%			226	23.35%			*p* = 0.018
Female	820	75.58%			78	66.67%			742	76.65%			
Race													
White	218	20.09%			26	22.22%			192	19.83%			*p* = 0.543
Black	593	54.65%			60	51.28%			533	55.06%			*p* = 0.438
Hispanic	274	25.25%			31	26.50%			243	25.10%			*p* = 0.743
Education													
≤High school graduate	413	38.06%			45	38.46%			368	38.02%			*p* = 0.925
Some college/technical school	450	41.47%			52	44.44%			398	41.12%			*p* = 0.490
College graduate	222	20.46%			20	17.09%			202	20.87%			*p* = 0.339
Employment													
Employed	731	67.37%			65	55.56%			666	68.80%			*p* = 0.004
Unemployed	354	32.63%			52	44.44%			302	31.20%			
Annual Household Income													
$0–≤ $14,999	269	24.79%			39	33.33%			230	23.76%			*p* = 0.024
$15,000–$34,999	307	28.29%			40	34.19%			267	27.58%			*p* = 0.134
$35,000–$49,999	229	21.11%			20	17.09%			209	21.59%			*p* = 0.260
$50,000 or higher	280	25.81%			18	15.38%			262	27.07%			*p* = 0.006
Rental assistance													
Used rental assistance	227	20.92%			34	29.06%			193	19.94%			*p* = 0.022
Did not use rental assistance	858	79.08%			83	70.94%			775	80.06%			
Marital status													
Married	496	45.71%			49	41.88%			447	46.18%			*p* = 0.378
Single	589	54.29%			68	58.12%			521	53.82%			
Household structure													
Number of children		1.16	1.28	0–4		1.27	1.34	0–4		1.15	1.27	0–4	*p* = 0.338
Number of adults		1.02	0.95	0–3		0.97	0.95	0–3		1.03	0.95	0–3	*p* = 0.551

^a^ Bivariate analyses were conducted to compare housing hardship measures and socio-demographic characteristics by individuals who experienced IPV victimization and those that did not. *p*-value for the significant comparisons are displayed. ^b^ During post-COVID-19 eviction moratoria—Harris: June 2020–December 2023; Travis: January 2022–December 2023. ^c^ Past 12 months.

**Table 2:** Covariate-adjusted logistic regression model for the association between housing instability domains and IPV, n = 1085.

	AOR	95% CI	*p*-Value
*Housing Instability*			
*Homelessness*			
Lifetime experience with homelessness	1.92	[1.61, 2.28]	*p* < 0.001
Never experienced homelessness	1.00	--	
Experienced eviction post-COVID-19 moratoria ^a^	0.74	[0.29, 1.92]	*p* = 0.539
Did not experience an eviction post-COVID-19 moratoria ^a^	1.00	--	
Housing mobility (i.e., # of moves) ^b^	0.86	[0.71, 1.03]	*p* = 0.106
Resided in shelter, abandoned building, car (i.e., unconventional space) ^b^	2.10	[1.92, 2.29]	*p* < 0.001
Never resided in an unconventional space ^b^	1.00	--	
Moved in with other people ^b^	1.20	[1.06, 1.36]	*p* = 0.005
Never moved in with other people ^b^	1.00	--	
*Lease Violations* ^b^			
Not pay full amount of rent	0.66	[0.35, 1.22]	*p* = 0.184
Paid full amount of rent	1.00	--	
Missed rent payments	1.69	[1.68, 1.71]	*p* < 0.001
Never missed rent payments	1.00	--	
Violated any part of lease (not related to payment)	2.50	[2.29, 2.73]	*p* < 0.001
Never violated any part of lease	1.00	--	
*Utility Hardship* ^b^			
Utilities turned off	1.62	[1.37, 1.91]	*p* < 0.001
Utilities never turned off	1.00	--	
*Poor Housing Quality* ^b^			
Poor living conditions	1.09	[0.94, 1.26]	*p* = 0.265
Unsafe housing environment	1.65	[1.31, 2.09]	*p* < 0.001
Safe housing environment	1.00	--	
*Sociodemographic Controls*			
Age	0.99	[0.97, 1.00]	*p* = 0.093
Sex			
Male	1.74	[0.94, 3.22]	*p* = 0.079
Female	1.00	--	
Race			
White	1.61	[1.15, 2.26]	*p* = 0.006
Black	1.00	--	
Hispanic	1.29	[0.93, 1.79]	*p* = 0.126
Education			
≤High school graduate	0.81	[0.44, 1.47]	*p* = 0.483
Some college/technical school	1.00	--	
College graduate	1.25	[0.92, 1.70]	*p* = 0.151
Employment			
Employed	1.00	--	
Unemployed	1.38	[0.96, 1.98]	*p* = 0.082
Annual Household Income			
$0- ≤ $14,999	1.05	[0.68, 1.62]	*p* = 0.825
$15,000-$34,999	1.31	[0.94, 1.82]	*p* = 0.107
$35,000-$49,999	0.97	[0.91, 1.02]	*p* = 0.206
$50,000 or higher	1.00	--	
Rental assistance			
Used rental assistance	1.42	[1.23, 1.64]	*p* < 0.001
Did not use rental assistance	1.00	--	
Marital status			
Married	0.97	[0.52, 1.83]	*p* = 0.935
Single	1.00	--	
Household structure			
Number of children	1.06	[0.92, 1.21]	*p* = 0.439
Number of adults	1.01	[0.75, 1.35]	*p* = 0.952

^a^ During post-COVID-19 eviction moratoria—Harris: June 2020–December 2023; Travis: January 2022–December 2023. ^b^ Past 12 months.

## Data Availability

The data presented in this article are not readily available because the data is part of an ongoing study.

## Abbreviations

AOR	Adjusted Odds Ratio
HITS Screener	Hurt, Insult, Threaten, Scream Screener
HOPE-M	Health Outcomes Post-Eviction-Moratoria
HPRM	Housing Precarity Risk Model
IPV	Intimate Partner Violence

## References

[1] World Health Organization Intimate Partner Violence. Violence Info 2022. https://apps.who.int/violence-info/intimate-partner-violence/.

[2] Leemis R.W., Norah F., Srijana K., May S.C., Marcie-jo K., Sharon G.S., Sharon C., Kathleen C.B. (2022). The National Intimate Partner and Sexual Violence Survey: 2016/2017 Report on Intimate Partner Violence.

[3] (2024). New Analysis Shows 8% Increase in U.S. Domestic Violence Incidents Following Pandemic Stay-At-Home Orders.

[4] (2025). Violence Against Women.

[5] Pavao J., Alvarez J., Baumrind N., Induni M., Kimerling R. (2007). Intimate partner violence and housing instability. Am. J. Prev. Med..

[6] Tsai J., Szymkowiak D., Jutkowitz E. (2022). Developing an operational definition of housing instability and homelessness in Veterans Health Administration’s medical records. PLoS ONE.

[7] Frederick T.J., Chwalek M., Hughes J., Karabanow J., Kidd S. (2014). How stable is stable? Defining and measuring housing stability. J. Community Psychol..

[8] Baker C.K., Cook S.L., Norris F.H. (2003). Domestic violence and housing problems: A contextual analysis of women’s help-seeking, received informal support, and formal system response. Violence Against Women.

[9] Benson-Goldsmith M.E., Gildea B., Richards T.N., Roley-Roberts M.E., Greenberg P., Hobbs A. (2025). An exploratory analysis of domestic and intimate partner violence victimization among persons experiencing eviction. Violence Against Women.

[10] Daoud N., Matheson F.I., Pedersen C., Hamilton-Wright S., Minh A., Zhang J., O’Campo P. (2016). Pathways and trajectories linking housing instability and poor health among low-income women experiencing intimate partner violence (IPV): Toward a conceptual framework. Women Health.

[11] Groves A.K., Smith P.D., Gebrekristos L.T., Keene D.E., Rosenberg A., Blankenship K.M. (2022). Eviction, intimate partner violence and HIV: Expanding concepts and assessing the pathways through which sexual partnership dynamics impact health. Soc. Sci. Med..

[12] Hargrave A.S., Knight K.R., Dhatt Z.K., Taylor G., Martinez D., Kushel M. (2025). The impact of intimate partner violence on homelessness and returns to housing: A qualitative analysis from the California statewide study of people experiencing homelessness. J. Interpers. Violence.

[13] Jasinski J.L., Wesely J.K., Mustaine E., Wright J.D. (2005). The Experience of Violence in the Lives of Homeless Women: A Research Report.

[14] Khanna M., Singh N.N., Nemil M., Best A., Ellis C.R. (1992). Homeless women and their families: Characteristics, life circumstances, and needs. J. Child Fam. Stud..

[15] Rollins C., Glass N.E., Perrin N.A., Billhardt K.A., Clough A., Barnes J., Hanson G.C., Bloom T.L. (2012). Housing instability is as strong a predictor of poor health outcomes as level of danger in an abusive relationship: Findings From the SHARE Study. J. Interpers. Violence.

[16] Klein L., Chesworth B.R., Howland-Myers J.R., Rizo C.F., Macy R.J. (2021). Housing interventions for intimate partner violence survivors: A systematic review. Trauma Violence Abus..

[17] Marçal K.E., Fowler P.J., Hovmand P.S., Cohen J. (2021). Understanding mechanisms driving family homeless shelter use and child mental health. J. Soc. Serv. Res..

[18] Schwab-Reese L.M., Peek-Asa C., Parker E. (2016). Associations of financial stressors and physical intimate partner violence perpetration. Inj. Epidemiol..

[19] Desmond M., Valdez N. (2013). Unpolicing the urban poor: Consequences of third-party policing for inner-city women. Am. Sociol. Rev..

[20] Raghavan C., Mennerich A., Sexton E., James S.E. (2006). Community violence and its direct, indirect, and mediating effects on intimate partner violence. Violence Against Women.

[21] Voith L.A., Brondino M.J. (2017). Neighborhood predictors of intimate partner violence: A theory-informed analysis using hierarchical linear modeling. Am. J. Community Psychol..

[22] Conger R.D., Elder Jr G.H., Lorenz F.O., Conger K.J., Simons R.L., Whitbeck L.B., Huck S., Melby J.N. (1990). Linking economic hardship to marital quality and instability. J. Marriage Fam..

[23] Fox G.L., Benson M.L., DeMaris A.A., Van Wyk J. (2002). Economic distress and intimate violence: Testing family stress and resources theories. J. Marriage Fam..

[24] Stark E. (2013). Coercive control. Violence Against Women.

[25] Arnold G. (2009). A battered women’s movement perspective of coercive control. Violence Against Women.

[26] Voth Schrag R.J., Ravi K.E., Robinson S.R. (2020). The role of social support in the link between economic abuse and economic hardship. J. Fam. Violence.

[27] Thomas T., Chapple K., Ramiller A., Phillips S., Campbell D., Greenberg J., Jessup B. The Housing Precarity Risk Model (HPRM): Predicting Evicition Risk Post-Pandemic. https://www.urbandisplacement.org/maps/housing-precarity-risk-model/.

[28] Hepburn P., Louis R., Fish J., Lemmerman E., Alexander A.K., Thomas T.A., Koehler R., Benfer E., Desmond M. (2021). US eviction filing patterns in 2020. Socius.

[29] Trestle Trestle. https://trestleiq.com/.

[30] Cosgrove J.A. (2018). Using a small cash incentive to increase survey response. Adm. Policy Ment. Health.

[31] Hawley K.M., Cook J.R., Jensen-Doss A. (2009). Do noncontingent incentives increase survey response rates among mental health providers? A randomized trial comparison. Adm. Policy Ment. Health.

[32] Acharya B., Bhatta D., Dhakal C. (2022). The risk of eviction and the mental health outcomes among the US adults. Prev. Med. Rep..

[33] Sherin K.M., Sinacore J.M., Li X.Q., Zitter R.E., Shakil A. (1998). HITS: A short domestic violence screening tool for use in a family practice setting. Fam. Med..

[34] StataCorp (2019). Stata Statistical Software.

[35] Mair C., Cunradi C.B., Todd M. (2012). Adverse childhood experiences and intimate partner violence: Testing psychosocial mediational pathways among couples. Ann. Epidemiol..

[36] Adams A.E., Beeble M.L. (2019). Intimate partner violence and psychological well-being: Examining the effect of economic abuse on women’s quality of life. Psychol. Violence.

[37] Council on Community Pediatrics & Community on Nutrition (2015). Promoting food security for all children. Pediatrics.

[38] Blau S.J., Tovar A., Pearlman D.N., Weeks H.M., Ali J., Bauer K.W. (2025). “It makes you feel worthless.” The lived experience of discrimination in the US food assistance system. Soc. Sci. Med..

[39] Bruckner H.K., Westbrook M., Loberg L., Teig E., Schaefbauer C. (2021). “Free” food with a side of shame? Combating stigma in emergency food assistance programs in the quest for food justice. Geoforum.

